# Pharmaceutical Notes

**Published:** 1909-05-29

**Authors:** 


### PHARMACEUTICAL NOTES.

### Artificial Calamine.

The question of the colouring of lotions, powders and ointments for dermatological practice is one to which sufficient importance is not always attached. For treatment of affections of the skin in the case of patients who are able to go about their ordinary business it is desirable that applications prescribed should be, as far as possible, invisible. Calamine was formerly used to a great extent because of its curative properties, and because its colour resembled to some extent that of the skin. This substance, however, was not included in the British Pharmacopœia of 1898, and since then practitioners have found it difficult to obtain calamine preparations of standard quality or colour. Professor R. B. Wild has recently been investigating this question, and gave the results of his experiments at the annual meeting of the British Pharmaceutical Conference which has just been held at Manchester. He recommends an artificial calamine composed of 2½ parts of Armenian bole and 97½ parts of precipitated zinc carbonate as a preparation which comes very near indeed to the colour of the normal skin. As preparations of this character are frequently required in dermatological practice it is desirable that a suitable formula should be devised and included in the Pharmacopœia.

